# Simple One-Step and Rapid Patterning of PDMS Microfluidic Device Wettability for PDMS Shell Production

**DOI:** 10.3389/fbioe.2022.891213

**Published:** 2022-04-19

**Authors:** Chunying Feng, Kohei Takahashi, Jianan Zhu

**Affiliations:** ^1^ Frontier Science Center for Synthetic Biology and Key Laboratory of Systems Bioengineering (Ministry of Education), School of Chemical Engineering and Technology, Tianjin University, Tianjin, China; ^2^ Graduate School of Life and Environmental Sciences, University of Tsukuba, Ibaraki, Japan

**Keywords:** microfluidics, surface modification, double emulsions, microcapsules, wettability

## Abstract

Double emulsion (DE) droplets with controlled size and internal structure are a promising platform for biological analysis, chemical synthesis, and drug delivery systems. However, to further “democratize” their application, new methods that enable simple and precise spatial patterning of the surface wettability of droplet-generating microfluidic devices are still needed. Here, by leveraging the increase in hydrophilicity of polydimethylsiloxane (PDMS) due to the plasma-treatment used to permanently bond to glass, we developed a one-step method to selectively pattern the wettability of PDMS microfluidic devices for DE generation. Our results show that both Aquapel-treated and 1H,1H,2H,2H-Perfluorodecyltriethoxysilan (PFDTES)-treated devices are functionally showing the generality of our method. With the resulting microfluidic devices, both water-in-oil-in-water (w/o/w) and oil-in-water-in-oil (o/w/o) DE droplets can be produced. Using a PDMS mixture containing cross-linking agents, we formed PDMS microcapsules by solidifying the shell layer of water-in-PDMS-in-water DE droplets. We also characterize the morphological properties of the generated droplets/microcapsules. We anticipate the method developed in this work could be used in a broad range of applications of DE droplets.

## Introduction

Droplet-based microfluidics have emerged as a promising tool due to its effective and exquisite control and operation (Jeong et al., 2021). The water-in-oil (w/o) droplets generated by microfluidic devices, which can be used as microreactors, have been widely applied in various fields, including pharmaceutical (Herranz-Blanco et al., 2014; Pessi et al., 2014; Kong et al., 2015), biochemical (Chen et al., 2009; Seiffert et al., 2010; Thiele et al., 2010; Abate et al., 2011; Mary et al., 2011; Clark et al., 2020), physics (Kanai et al., 2010; Choi et al., 2019; Shi et al., 2020), food (Lu et al., 2016; Zhang et al., 2016), cosmetic (Li et al., 2018; Sun et al., 2020, 2021; Jeong et al., 2021), agriculture (Chu et al., 2007; Neethirajan et al., 2011), and in genetic screening (Macosko et al., 2015; Zhang et al., 2015; Wang et al., 2020). However, the process of microdroplet formation is often accompanied by satellite small droplets either generated in flow-focusing orifices or separated from target droplets by spontaneously emulsification due to mechanical breakup or chemical instability (Schmitt et al., 2017; Zabar et al., 2020). Such instabilities of single microdroplets may cause cross contamination between dispersed phase and continuous phase, making it difficult to be applied in long-term analysis of single cell and targeted deliveries in controlled environments (Chen et al., 2011; Choi et al., 2013).

Double emulsions (DE) droplets overcome the aforementioned problems since the inner phase and the external environment are isolated through a core-shell structure (Hou et al., 2017; Wu et al., 2020; Jeong et al., 2021), which is formed by having one type of droplet dispersed in a lager immiscible droplet. As both the inner core and the shell structure can be separately controlled, DE droplets have been proven to be very useful for single-cell analysis (Moore et al., 2018; Zhang et al., 2018; Mutafopulos et al., 2020), transport of active and ions (Xie et al., 2017; Chowdhury et al., 2019; Pei et al., 2019; Sun et al., 2020; Wu et al., 2020; Jeong et al., 2021), functional microparticles (Kim et al., 2014), and particularly, fabrication of microcapsules through the gelation of the shell structure.

Droplet generator devices, particularly those made using soft-lithography (Xia and Whitesides, 1998), are usually made from polydimethylsiloxane (PDMS) due to the numerous advantages of this material, which include ease-of-use, low cost, optically transparent, non-toxic, and permeability of gases. However, due to the inherent hydrophobic nature of native PDMS surfaces, to generate DE droplets, the surface wettability of PDMS microfluidic devices must be selectively and spatially patterned to obtain distinct hydrophilic and hydrophobic surfaces.

A variety of methods have been developed to modify the channel wettability for DE droplets production, such as silane treatment (Nawar et al., 2020; Shi et al., 2020), sol–gel coatings (Abate et al., 2008), plasma and UV treatment (Bai et al., 2015; Brower et al., 2020; Kong et al., 2020), layer-by-layer (LBL) deposition (Choi et al., 2018), chemical grafting/deposition combined with plasma treatment (Trantidou et al., 2017), and flow-blocking of particular zones by epoxies or resins (Bai et al., 2015; Li et al., 2016; Bodin-Thomazo et al., 2017). Although all these surface-treatment methods are effective, they are often time-consuming and cumbersome to apply and may change the dimensions of the designed microfluidic channels. For instance, the “marker technique” modifies PDMS wettability by partially blocking certain channel zones but leads to an altered channel size (Bodin-Thomazo et al., 2017). Therefore, in this field, there is still a need for the development of simpler and more efficient ways to pattern the surface wettability of microfluidic devices in a precisely and spatially controllable manner for DE droplets generation. In addition, most of the aforementioned surface-treatment methods focus on modifying the second flow-focusing junction by changing the outermost (continuous phase) channel to be hydrophilic to enable water-in-oil-in-water (w/o/w) DE droplets. Conversely, there are relatively few methods that focus on modifying the first flow-focusing junction, where the internal droplet is generated.

In this study, we present a simple, one-step, rapid, and effective surface-treatment strategy based on flow confinement for DE droplets generation, which enables precise chemical patterning of the substrate surface. Our method takes advantage of the air plasma treatment executed during the fabrication process of devices to obtain the hydrophilic surface. It requires one additional step to convert the desired junction from hydrophilic to hydrophobic. Two different surface modification agents, Aquapel and Perfluorodecyltriethoxysilan (PFDTES), were tested. Our microfluidic devices maintain their surface properties and functionality for at least 10 days if they are stored in water and can successfully generate both w/o/w and o/w/o DE droplets varieties. Using unpolymerized PDMS containing a reticulant agent (cross-linking agent existed in Sylgard 184 elastomer kit) as the middle oil phase, microcapsules were also obtained after the solidification of the PDMS shell, yielding water-in-PDMS-in-water (w/PDMS/w) droplets.

## Materials and Methods

### Materials

Mineral oil (330779), polyvinyl alcohol (PVA, 87–89% hydrolyzed, average Mw 1/4 13,000–23,000) were obtained from Sigma-Aldrich. Deionized water (DI-water) was purified by an ultrapure Deionized water system (HYP-QX, China). PDMS (Sylgard 184 elastomer kit) and PMX-200 were purchased from Dow corning. Sorbitan monolaurate (Span 80) was obtained from Shanghai YuanYe Biotechnology. Aquapel was purchased from PPG Industries, while 1H,1H,2H,2H-PFDTES was purchased from Shanghai Macklin Biochemical Co., Ltd. All aqueous reagents were filtered with a 0.22 *μ*m filter membrane (Millipore).

### Chip Fabrication

**FIGURE 1:**
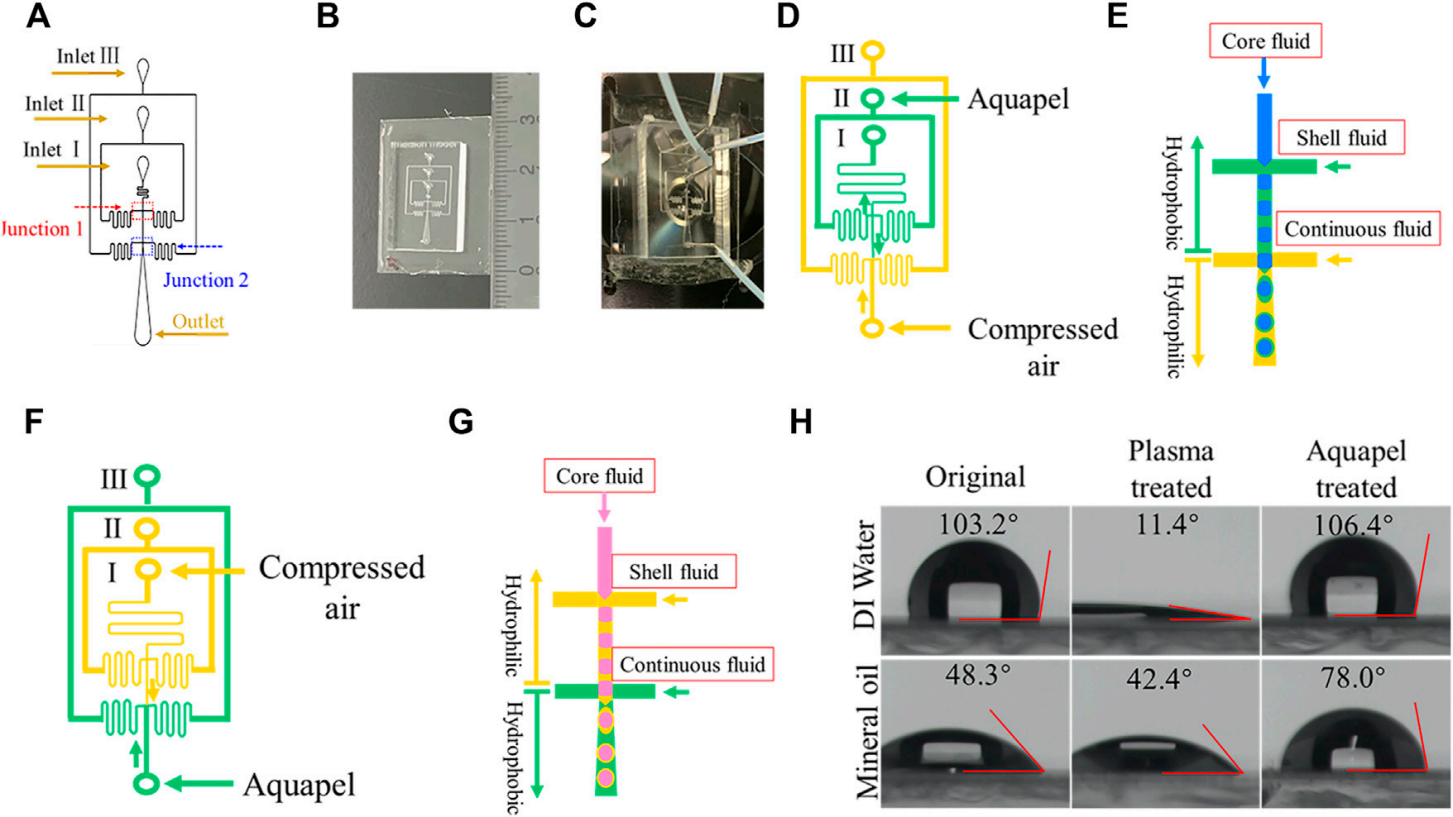
Chip design and microfluidic channel surface modification. **(A)** Schematic diagram showing the layout of the microfluidic device. The looping channels serve to increase flow resistance. **(B)** Photograph showing the size of a PDMS device. **(C)** Photograph of a PDMS device connected with tubing. **(D)** Hydrophobic modification with Aquapel of designated channels (green) for forming w/o/w DE droplets. The arrows indicate the direction of liquid flow. **(E)** Schematic illustration of w/o/w DE droplets generation process. **(F)** Hydrophobic modification with Aquapel of designated channels for forming o/w/o DE droplets. **(G)** Schematic illustration of o/w/o DE generation process. **(H)** Contact angle (*θ*) of water and mineral oil droplets on an untreated PDMS surface, after plasma treatment, and after Aquapel treatment, respectively.

The wafer mold with two flow-focusing orifices was manufactured by standard photolithography (Xia and Whitesides, 1998). A schematic diagram of the microfluidic device is shown in Figures 1A–C. The PDMS and curing agent (Sylgard 184 elastomer kit) were thoroughly mixed at a ratio of 10:1 and degassed. Then, the mixed solution was poured into the top of the patterned silicon wafer mold and degassed. The mixture was cured at 30°C in a vacuum oven for 24 h. Subsequently, the hardened PDMS was cut, peeled off the mold, and placed on aluminum foil. Biopsy punches (0.5 mm) were used for making inlets and outlet holes in the PDMS layer, which were then cleaned with 3M Scotch tape to remove any residue and dust behind. Glass slides soaked in ethanol were first treated by ultrasound for 10 min and then baked for 10 min at 80°C to dry. Finally, the PDMS layer was bonded to a glass slide after air plasma treatment of both the PDMS slab and glass slide using plasma cleaner (PDC-MG, MingHeng) (the parameters showed in Supplementary Table S1). In this work, unless otherwise noted, the parameters for plasma treatment to bond the microfluidic chip and glass slides are 136 W for 45 s.

### Surface Modification

To generate w/o/w DE droplets, specific channels must be modified to be hydrophobic to form w/o droplets, and other sections must be hydrophilic for generating o/w droplets. The surface treatment process workflow is shown in Figure 1. Since plasma treatment renders PDMS surfaces hydrophilic, we need only modify the first flow-focusing junction to be hydrophobic. To do so, we gently infuse Aquapel (or PFDTES solution) from the middle port inlet (labeled inlet II in Figure 1A) into the main channel by creating negative pressure by pulling on the plunger of a syringe connected via tubing to inlet I. Simultaneously, compressed air, connected *via* tubing directly to the outlet, is used to protect the second junction from invasion by Aquapel or PFDTES solution. The injection process takes 30–60 s. Finally, to clean the channels, we disconnect all connections other than the compressed air and then increase the pressure to expel residual Aquapel (or PFDTES solution) from the channels. Generally, the microfluidic device was prepared in the process of sample preparation due to the rapid treated process and used within 6 h.

Similarly, to generate o/w/o DE droplets, the hydrophilicity of the first junction must be maintained for producing o/w droplets, while the second junction needs to be modified to be hydrophobic for producing w/o droplets. In this case, Aquapel (or PFDTES) was infused through the outlet while compressed air was flown from inlet I to guide the Aquapel to flow out of inlet III (Figure 1) for 30–60 s. Then, Aquapel was expelled from the channels by increasing compressed air pressure. In this study, the devices were used immediately after this step.

### Double Emulsion Droplets Generation

DE droplets were produced using 3 syringe pumps (TYD01, Leadfluid) for loading the inner, middle, and outer phases. Each phase was loaded into plastic syringes (plastipak, BD) connected to PTFE tubing (ID 0.6 mm, OD 1.0 mm), which are coupled to the inlets on the PDMS chip *via* 15 mm long pieces of 21 Gauge metal tubing (ID 0.5 mm, OD 0.7 mm). The flow rates of the inner, middle, and outer phases, Q_*i*_, Q_*m*_, and Q_*o*_, were adjusted to achieve stable DE droplets.

To generate solidified PDMS microcapsules, the w/PDMS/w DE droplets were prepared, which begin as w/o/w droplets. The inner phase is PBS containing 5% w/v glycerol and 1% w/v PVA; the middle phase is a mixture of PDMS and silicone oil (50 cSt, PMX-200, Dow Corning) in a weight ratio of 3:7. To solidify the PDMS layer, we add a reticulating agent (10% w/w) to the oil mixture. The continuous aqueous phase is PBS with 5% w/v PVA. To ensure that each fluid reaches the junctions at approximately the same time, which stabilizes the process, we set the flow rates of the three phases to be 300 *μ*L/h; once the fluids reach the channels, we then change the flow rates to the desired values. The generated DE droplets were collected in a 0.5% w/v PVA solution. To measure the average value of the inner and outer diameter of DE droplets, we analyze at least 60 droplets.

To generate o/w/o DE droplets, the inner phase is a mineral oil containing 1% span 80, the middle phase is PBS containing 10% PVA, and the outer phase is a mineral oil containing 4% span 80. The generated DE droplets were collected in a mineral oil containing 0.5% w/v span 80.

### Rheological Measurements

We determined the rheological properties of different liquids used in these experiments with a TA Instruments HR-2 (hybrid rheometer). The geometry of the plate was a 25-mm Peltier stainless steel parallel plate. All experiments were performed using a gap of 900 *μ*m and a trim offset of 50 *μ*m. For gel-point experiments, the environmental temperature was maintained at 25°*C*. The percent strain and angular frequency selected are 4% and 10.0 rad/s.

### Image Capture

Images and movies of DE droplets were captured and recorded on an inverted microscope (Nikon, Japan; Leica, Germany) equipped with a Phantom V2512 high-speed camera (Vision Research, Wayne, NJ, United States). Images of microcapsules were captured by an sCMOS camera (Andor neo-5.5). Images were analyzed using ImageJ to characterize the sizes of DE droplets and microcapsules.

## Results and Discussion

### Device Design and Fabrication

The microfluidic channel of a device consists of two consecutive flow-focusing junctions for preparing w/o and w/o/w droplets (or inverse droplets), as shown in Figures 1A,B. We incorporate flow resistances into our designs to aid in stabilizing the fluid flow rate as well as reducing the possibility of backflow into the dispersed-phase channel or continuous-phase channel from flow-focusing junctions (Ho et al., 2016; Choi et al., 2018). To infuse the different phases, we connect tubing attached to syringes, as shown in Figure 1C.

### Surface Wettability Patterning With Aquapel

For the generation of DE droplets, it is critical to pattern the surface wetting properties of channels selectively and spatially (Choi et al., 2018; Trantidou et al., 2017; Kim et al., 2015). Each junction can be modified to be either hydrophobic for w/o droplet generation or hydrophilic for o/w droplet generation (Choi et al., 2019; Sun et al., 2020). Taking the generation of w/o/w droplets as an example, the heat-treatment has been applied in conventional methods to make the first junction hydrophobic (Trantidou et al., 2017), which causes the whole device to become hydrophobic (Supplementary Figure S1). In this case, after heat-treatment for several hours, the second junction must be selectively treated to become hydrophilic by another method as mentioned in the introduction (Lillehoj and Ho, 2010; Trantidou et al., 2017; Choi et al., 2018). However, these methods are complicated and time-consuming. In addition, ink, tape, or epoxy can be used to protect the hydrophobic of channel (Kim et al., 2015; Li et al., 2016). However, it can change the size of a microfluidic channel. Therefore, it is still needed to develop new DE device modification methods.

As stated in the fabrication process, before a PDMS replica is bonded to a glass slide, it must be plasma-treated to generate an activated surface (Tian et al., 2018). Meanwhile, this plasma treatment also renders the surface hydrophilic. Unlike the aforementioned conventional methods, only one additional step is required to selectively make the specific junction hydrophobic by leveraging this required treatment. Once completed, this will then fulfill the surface wettability requirements for generating DE droplets. For example, to fabricate w/o/w droplets, the first flow-focusing junction must be hydrophobic. Toward this goal, we injected Aquapel through inlet II and expelled it from inlet I manually to treat the inner and middle channels (see *Methods*). Simultaneously, we protected the outer channel from contact with Aquapel by injecting compressed air, which thus maintains its hydrophilicity (Figures 1D,E). To validate the hydrophobicity of the Aquapel-treated PDMS channels, we measured the contact angle *θ* of the original untreated, plasma-treated, and Aquapel-treated PDMS surfaces by the pendant drop method. The results are shown in Figure 1H. The untreated PDMS surface exhibits *θ*_water_ = 103.2° (*θ*_oil_ = 48.3°), which decreases to 11.4° (*θ*_oil_ = 42.3°) immediately after plasma treatment. After Aquapel treatment, the *θ*_water_ of the treated surface returns to 106.4° (*θ*_oil_ = 78.0°), indicating that the surface treatment methods are effective. Similarly, o/w/o droplets can be fabricated if the second junction is modified to be hydrophobic by Aquapel (Figures 1F,G). Compared with the aforementioned conventional methods, this method only requires one step to accomplish the surface modification while being simpler and easier to apply.

### Long-Term Hydrophilic Stability of Plasma-Treated Polydimethylsiloxane Surface

**FIGURE 2:**
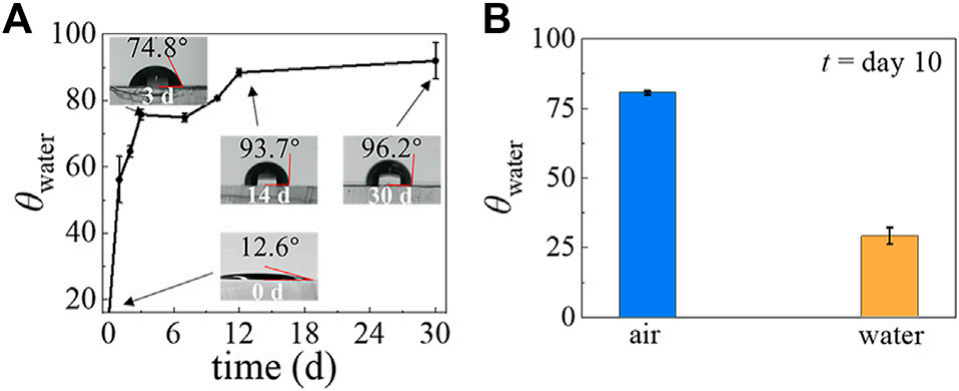
The temporal evolution of PDMS surface hydrophilicity after plasma treatment when stored in air and when stored in water. **(A)** The *θ*_water_ of the PDMS surface stored in ambient laboratory conditions, measured at different time points. **(B)** The *θ*_water_ of the PDMS surface after storage in air and in water for 10 days.

It has been shown that the PDMS surface can recover its hydrophobicity within hours after plasma treatment, but the exact recovery time varies (Lee et al., 2003). To better control the wettability of the PDMS device, we studied the time evolution of the hydrophilicity of modified PDMS surfaces. After a PDMS slab was treated by air plasma, we measured the water contact angle *θ*_water_ as a function of time, as shown in Figure 2A. We find that the *θ*_water_ of the plasma-treated PDMS surface stored in the air is 17.4° on day 1 but quickly increases to 74.9° by day 3. After that, the *θ*_water_ still increases but more slowly and reaches 93.7° on day 14. Beyond this point, the *θ*_water_ maintains a constant and stable value out to day 30. These results indicate that the hydrophobicity recovers quickly at the beginning, then reaches a plateau at a much slower rate. In addition, it is known that the *θ*_water_ of plasma-treated PDMS surfaces are also affected by the environment in which they are stored (Tian et al., 2018; Nguyen et al., 2014; Yang et al., 2012; Zhao et al., 2012; Liu et al., 2021). To investigate this point, we compared the *θ*_water_ of plasma-treated PDMS surfaces when they are stored in water and the air (Figure 2B). After a 10-days incubation, the *θ*_water_ of the plasma-treated PDMS is 29° when stored in water, whereas it is 80.7° when stored in the air. It indicates that the hydrophilicity of plasma-treated PDMS can be prolonged by storage in an aqueous environment, which agrees with previous results (Liu et al., 2021).

### Characterization and Optimization of Aquapel-Treated Time

**FIGURE 3:**
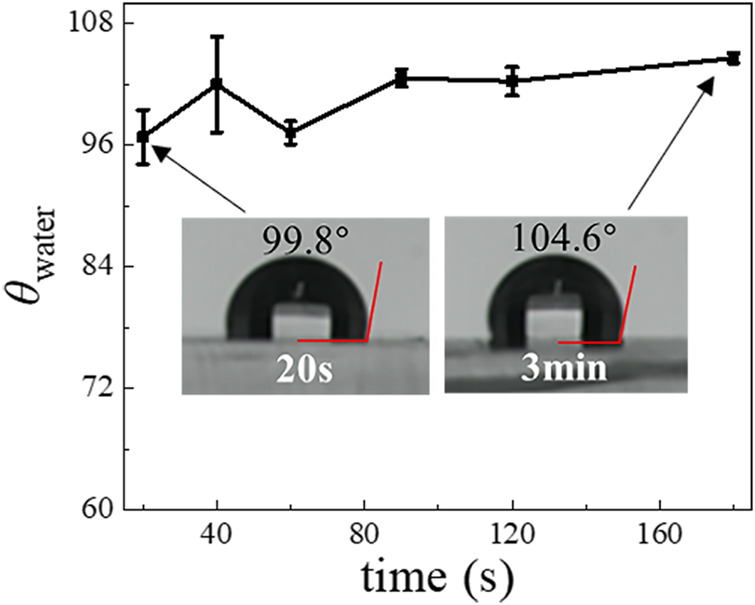
The *θ*_water_ of a PDMS surface with different Aquapel-treatment times.

To optimize the Aquapel-treatment, we further investigated the dependence of the resultant hydrophobicity of PDMS surfaces on treatment time. The results for the *θ*_water_ obtained by treating the plasma-treated PDMS surface using Aquapel for 20, 40, 60, 90, 120, and 180 s, respectively, are shown in Figure 3. Although we find that the *θ*_water_ slightly increases from 97° to 105° when we increase treatment time, a 20 s application time already appears sufficient to achieve a large change in hydrophobicity. Compared with heating methods, the Aquapel-treatment is relatively fast and can save time. We also tested the stability of the Aquapel-treated PDMS surface, and the results show that it maintains its modified hydrophobicity in the air for at least several weeks (Supplementary Table S2).

### Generation of Polydimethylsiloxane Shell With a Controllable Size and Morphology

**FIGURE 4:**
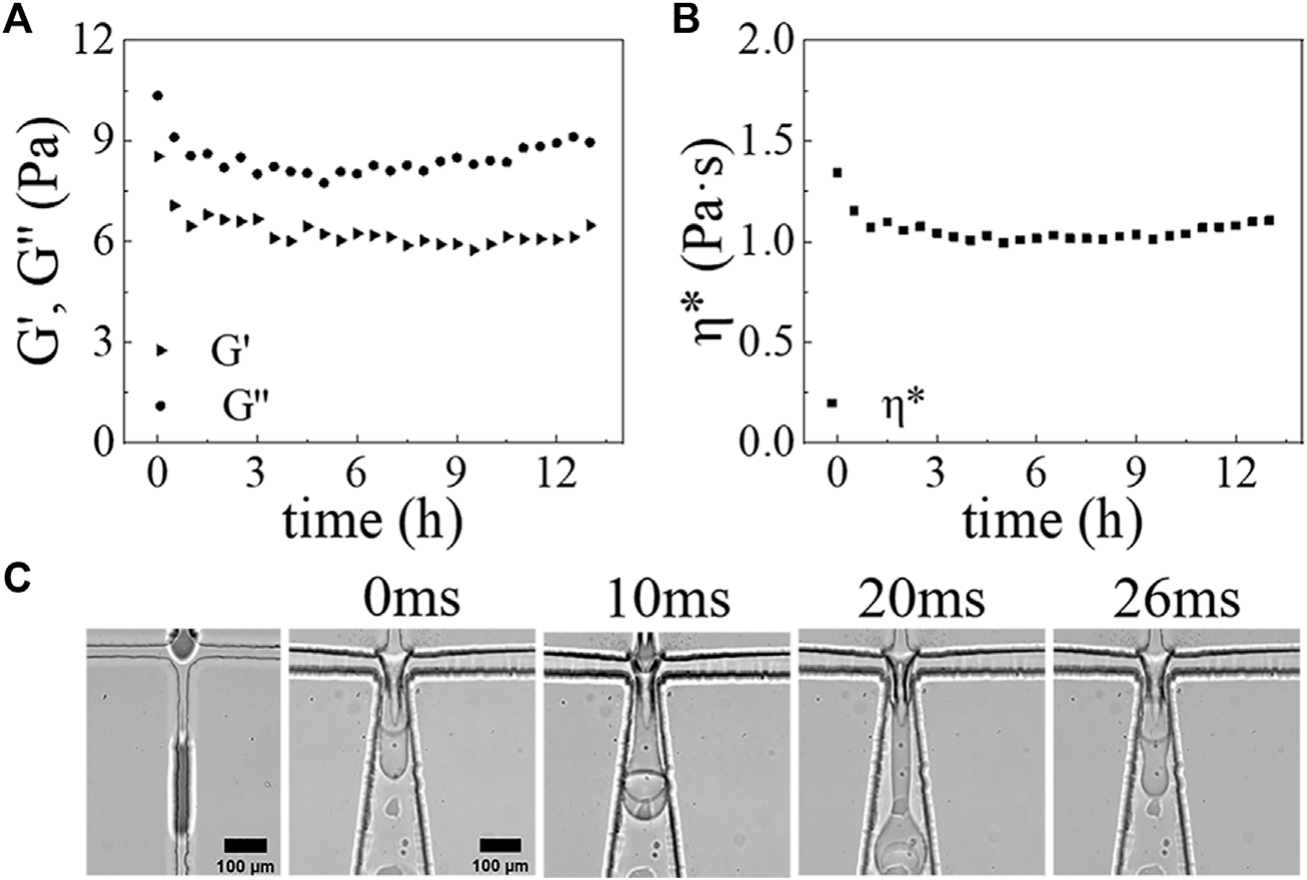
The rheological properties of PDMS mixture versus time at 25°C: **(A)** Storage modulus (G’) (triangular), loss modulus (G”) (circular) of PDMS mixture versus time. **(B)** Complex viscosity (η*) (square) of PDMS mixture versus time. **(C)** Optical micrograph images showing the formation process of DE droplets at the first junction and second junction at 0, 10, 20, and 26 ms. Scale bars are 100 *μ*m.

To test the effectiveness of our surface modification method, we employ the fabricated PDMS device to produce w/PDMS/w DE droplets that are subsequently polymerized to form microcapsules. We find that the cross-linking of PDMS is a very slow process under the conditions of DE droplets generation. Bulk rheological measurements of the middle oil phase show very little change in the loss and storage moduli within 6 h after adding cross-linking agents (Figures 4A,B). This plateau indicates that the solidification of the mixture is not significant within the first 6 h, during which the w/PDMS/w DE droplets are produced. It also indicates that we can neglect the effect of changes in viscoelasticity of the middle phase on the generation of DE droplets due to any cross-linking that may begin during drop generation. As expected, our devices generate w/PDMS droplets in the first junction, which are then encapsulated in the outer aqueous phase, forming a PDMS/w droplet in the second junction (Figure 4C, Supplementary Videos S1–S3). The successful generation of the w/PDMS/w DE droplets indicates that the surface modifications are successful and robust. To better validate the efficiency and performance of the surface modification method, we also investigated the DE droplets formation in the microfluidic chip with or without Aquapel treatment. For w/PDMS/w DE droplets generation in the microfluidic device without Aquapel treatment, we find the w/PDMS droplets are generated only in the first junction, and no new droplets are generated in the second junction, indicating the second junction is not hydrophobic and could not generate w/PDMS droplets (Supplementary Figure S2, Supplementary Video S4). In addition, for w/PDMS/w DE droplets generation in a microfluidic device treated completely by Aquapel, we find no droplets are generated in the first junction, and the PDMS/w droplets are generated in the second junction, indicating the first junction is not hydrophilic and could not generate PDMS/w droplets (Supplementary Figure S3, Supplementary Video S5). These results fully confirm plasma treatment could make the microfluidic channel hydrophilic, and Aquapel could selectively modify the surface of the plasma-treated microfluidic channel from hydrophilic to hydrophobic. To test the long-term reliability of the modified method for DE droplets, we made the modified microfluidic device store in a water environment for 10 days. After that, this microfluidic device was used for DE droplets generation. The results show the modified microfluidic device could still reliably generate w/PDMS/w droplets (Supplementary Videos S6, S7).

**FIGURE 5:**
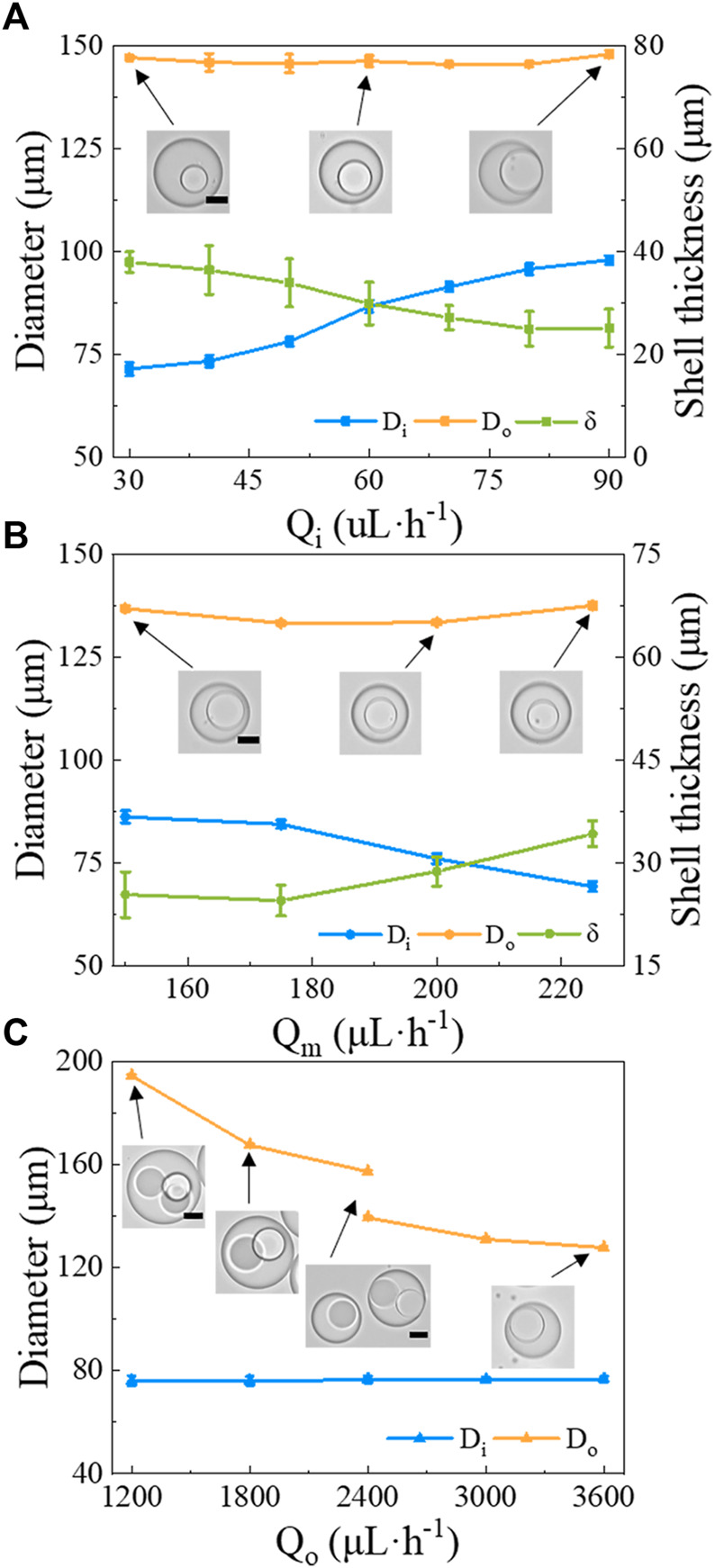
The morphology of w/PDMS/w DE droplets versus flow rates of the different phases. The diameter of the core (D_*i*_), the diameter of the DE droplet (D_*o*_), and the shell thickness *δ* are shown under the following conditions: **(A)** varying Q_*i*_, while keeping Q_*m*_ = 200 *μ*L/h and Q_*o*_ = 3,000 *μ*L/h; **(B)** varying Q_*m*_, while Q_*i*_ = 50 *μ*L/h and Q_*o*_ = 3,000 *μ*L/h; **(C)** varying Q_*o*_, while Q_*i*_ = 50 *μ*L/h and Q_*m*_ = 200 *μ*L/h. Scale bars in the insets represent 50 *μ*m.

To clarify how to control over the DE droplets morphologies, we further investigated the effect of flow rates of different phases on the morphology of DE droplets. When we increase Q_*i*_ from 30 to 90 *μ*L/h while keeping Q_*m*_ = 200 *μ*L/h and Q_*o*_ = 3,000 *μ*L/h, the diameter of the inner droplet D_*i*_ also increases from 71.4 ± 1.7 *μ*m to 97.8 ± 1.1 *μ*m, while the diameter of the DE droplets D_*o*_ remains roughly constant (∼ 145.0 ± 2.3 *μ*m). Thus, the shell thickness, defined as *δ*=(D_*o*_-D_*i*_)/2, decreases from 37.8 *μ*m to 25 *μ*m as shown in Figure 5A. Similarly, when Q_*m*_ is increased from 150 to 225 *μ*L/h while Q_*i*_ = 50 *μ*L/h and Q_*o*_ = 3,000 *μ*L/h, D_*i*_ is reduced from 86.1 ± 1.3 *μ*m to 69.2 ± 0.8 *μ*m while D_*o*_ is roughly kept constant (∼ 135.2 ± 2.2 *μ*m), which then results in an increase of *δ* from 25.3 *μ*m to 34.2 *μ*m (Figure 5B). By contrast, when Q_*o*_ increases from 1,200 to 3,600 *μ*L/h while Q_*i*_ = 50 *μ*L/h and Q_*m*_ = 200 *μ*L/h, the structure of DE droplet is changed dramatically. At Q_*o*_ = 1,200 *μ*L/h, large droplets that each contain three smaller core droplets are produced. As Q_*o*_ increases, the number of core droplets decreases. For example, at Q_*o*_ = 1,800 *μ*L/h, large droplets that contain two smaller cores are observed. When Q_*o*_ ≥ 2,400 *μ*L/h, DE droplets with a single core droplet are obtained (Figure 5C). During the tested range of Q_*o*_, D_*i*_ is independent of Q_*o*_ and is around 76 *μ*m, whereas D_*o*_ decreases from 194.5 ± 0.9 *μ*m (Q_*o*_ = 1,200 *μ*L/h) to 127.8 ± 0.9 *μ*m (Q_*o*_ = 3,600 *μ*L/h) with a relatively large reduction when the number of cores decreases (Figure 5C). Taken together, these results suggest that Q_*o*_ largely affects D_*o*_ and number of core droplets, while Q_*i*_ and Q_*m*_ strongly affect D_*i*_.

**FIGURE 6:**
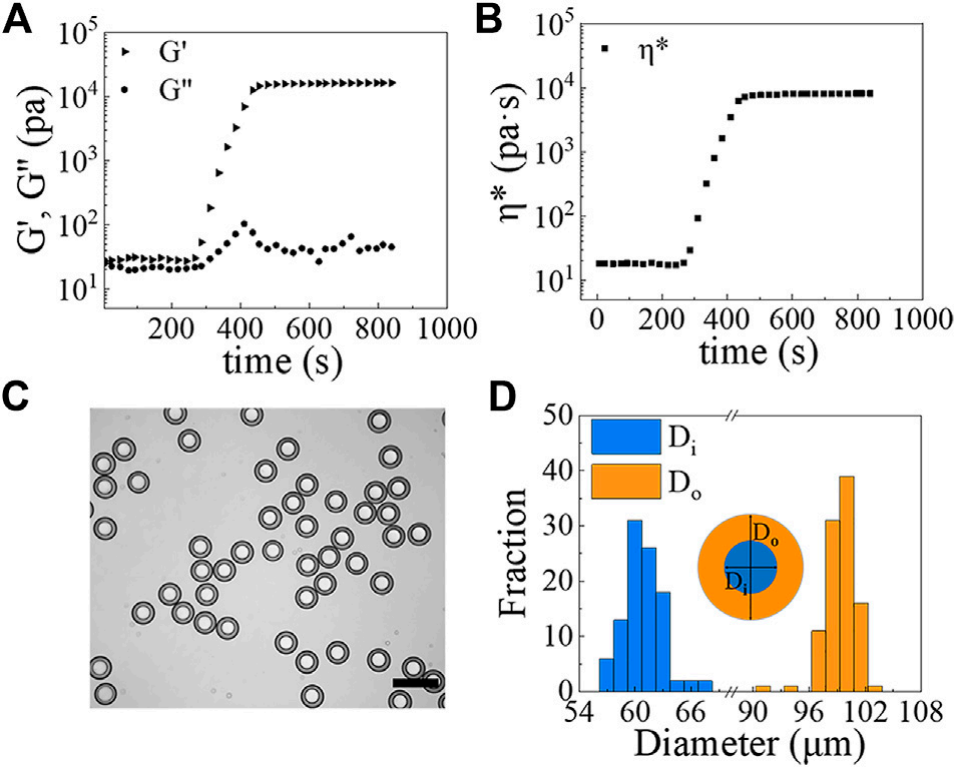
**(A,B)** The rheological properties of the PDMS mixture versus time at 80°C: **(A)** G’ (triangle), G” (circle); **(B)** η* (square). **(C)** Image of PDMS microcapsules formed from w/PDMS/w DE droplets. Scale bar represents 200 *μ*m. **(D)** Size measurements of microcapsules (N ≥100).

With the w/PDMS/w DE droplets in hand, microcapsules can be formed by allowing the PDMS layer of DE droplets to solidify. To speed solidification, we collected the DE droplets in a 0.5% w/v PVA solution and heated them in an oven at 80°C for 1.5 h. The rheological measurements of the PDMS mixture incubated at 80°C as a function of time shows that it hardens within 10 min (Figures 6A,B). Thus, the 1.5 h incubation time at 80°C is sufficient to solidify the shell layer of w/PDMS/W DE droplets. An example of shell-solidified droplets (microcapsules) generated under the conditions of Q_*i*_ = 50 *μ*L/h, Q_*m*_ = 200 *μ*L/h and Q_*o*_ = 4,800 *μ*L/h is shown in Figure 6C. The diameters of DE droplets change very little over the course of shell-solidification. Prior to solidification D_*i*_ = 57.6 ± 0.9 *μ*m and D_*o*_ = 96.5 ± 1.2 *μ*m (N > 100), whereas after solidification D_*i*_ = 60.8 ± 2.1 *μ*m and D_*o*_ = 97.9 ± 1.7 *μ*m. Our microcapsules have a narrow size distribution with a polydispersity of 3.5% (C.V.) in D_*i*_ and 1.7% (C.V.) in D_*o*_ (Figure 6D).

### Generation of o/w/o Double emulsion Droplets With Aquapel-Treated Microfluidic Chip

**FIGURE 7:**
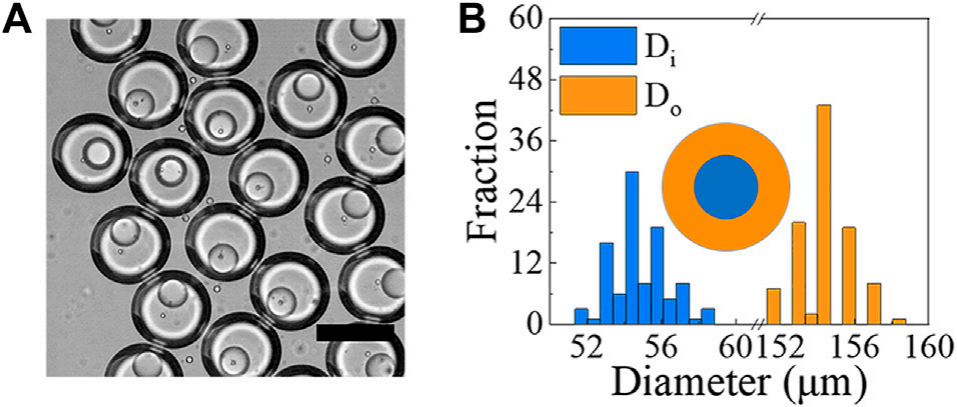
**(A)** Optical image showing examples of o/w/o droplets generated using microfluidic devices. Core phase is mineral oil with 1% span 80, while shell phase is 10% PVA in PBS and outer phase is mineral oil with 4% span 80. Scale bar represents 150 *μ*m. **(B)** Size measurements of o/w/o droplets generated under the conditions of Q_*i*_ = 30 *μ*L/h, Q_*m*_ = 500 *μ*L/h and Q_*o*_ = 1,500 *μ*L/h (N ≥ 100).

Similarly, when the surface wettability of the microfluidic device is appropriately modified (Figures 1F,G), we generate o/w/o DE droplets, as described in the *Methods* section. For this type of DE droplets, the o/w emulsions are generated in the first flow-focusing junction, which are then encapsulated into a bigger droplets to form the o/w/o DE droplets in the second junction (Figure 7A for images and drop generation in Supplementary Videos S8–S10). Using the following flow rates: Q_*i*_ = 30 *μ*L/h, Q_*m*_ = 500 *μ*L/h and Q_*o*_ = 1,500 *μ*L/h, we generate DE droplets with D_*i*_ = 54.5 ± 1.5 *μ*m (C.V. = 2.7%) and D_*o*_ = 154.0 ± 1.4 *μ*m (C.V. = 1%), as shown in Figure 7B.

### Characterization and Optimization of PFDTES-Treated Polydimethylsiloxane Surface

**FIGURE 8:**
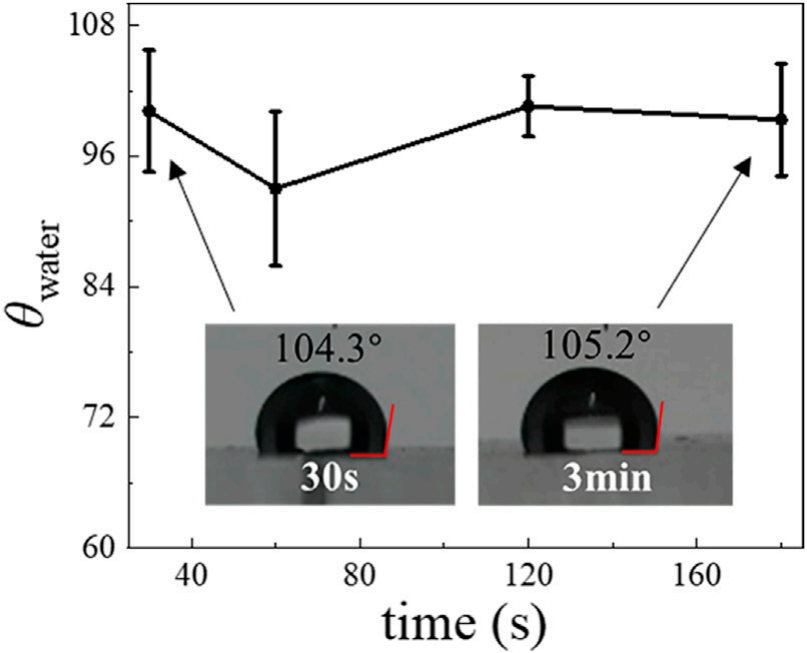
The *θ*_water_ of PDMS surface with different PFDTES—treatment time.

To show the generality and utility of our surface modification method to prepare DE droplets, we tested a commonly available fluorinated silane, PFDTES. The *θ*_water_ of plasma-treated PDMS surfaces becomes ∼ 100° after PFDTES treatment for 30–180 s (Figure 8), showing that PFDTES-treatment produces a similar result as does with Aquapel to render the surface hydrophobic. We then replaced Aquapel with PFDTES and repeated the steps shown in Figure 1E to generate DE droplets (Figure 1G).

### Generation of Polydimethylsiloxane Shell With PFDTES-Treated Microfluidic Chip

**FIGURE 9:**
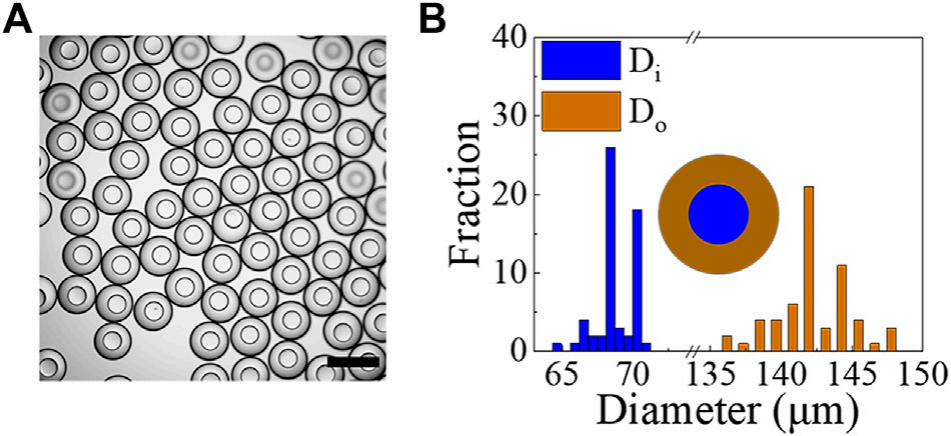
**(A)** Optical image showing examples of microcapsules obtained from w/PDMS/w droplets using microfluidic devices whose channel surfaces were treated with PFDTES. Scale bar represents 200 *μ*m. **(B)** Size measurements of microcapsules generated under the conditions of Q_*i*_ = 10 *μ*L/h, Q_*m*_ = 200 *μ*L/h and Q_*o*_ = 600 *μ*L/h (N ≥60).

These modified PDMS devices are tested to generate w/PDMS/w DE droplets. The DE droplets generated under the conditions of Q_*i*_ = 10 *μ*L/h, Q_*m*_ = 200 *μ*L/h and Q_*o*_ = 600 *μ*L/h, are shown in Figure 9A and Supplementary Videos S11, S12. We solidified the microcapsules as described previously by incubating them at 80°C for 1.5 h. The as-obtained microcapsules have a narrow size distribution with Di = 68.4 ± 1.3 *μ*m (C.V. = 2.0%) and Di = 142.0 ± 2.6 *μ*m (C.V. = 1.9%) (averaged over 60 microcapsules) (Figure 9B). We also tested the stability of the PFDTES-treated PDMS surface. The results show that it maintains its modified hydrophobicity in air for at least several weeks (Supplementary Table S2).

## Conclusion

Spatial and selectively control of wettability is necessary for DE droplets generation devices. Numerous researchers have successfully generated DE droplets in PDMS microfluidic devices by modifying the wetting properties within the channels. Our aim in this work is to develop a simple, single-step, and rapid method to pattern the wettability of PDMS microfluidic devices to simplify DE droplets generation. The method we describe leverages the plasma treatment required for bonding the PDMS replica with glass substrates by utilizing the fact that it also renders the whole PDMS chip surface hydrophilic. We selectively coat certain channels with Aquapel (or PFDTES) while blocking its flow into undesired regions with compressed air in a short treatment (30–60 s), which leaves the inner and middle channels as well as the first junction hydrophobic. Meanwhile, the outer channel and the second junction are hydrophilic; this patterning readily allows us to generate w/o/w DE droplets. We show that the resulting devices maintain their surface wetting properties for at least 10 days when stored submerged in water, with water filling the microfluidic channels. To our best knowledge, methods mentioned in the section “Surface Wettability Patterning with Aquapel” are generally time-consuming and involve complicated operations. Compared with these methods, our surface modification method, which takes seconds to implement, achieves the same wetting properties but with a much simpler protocol. The simplicity of this method makes it possible to simultaneously pattern the surface wettability in a large number of devices. We are also able to easily generate the less-frequently used inverse o/w/o DE droplets. In this case, we reverse the wetting properties of microfluidic channels. We hope that the detailed descriptions in this study may provide a point of reference and anticipate this method will enable easier adoption of DE droplets into a broad range of applications.

## Data Availability

The original contributions presented in the study are included in the article/Supplementary Material, further inquiries can be directed to the corresponding author.
