# Supplementary material for: Simple One-Step and Rapid Patterning of PDMS Microfluidic Device Wettability for PDMS Shell Production
Source: Front Bioeng Biotechnol. 2022 Apr 19;10:891213. doi: 10.3389/fbioe.2022.891213 (PMC9061991; doi:10.3389/fbioe.2022.891213)
Supplement: Supplementary file 5 [file DataSheet1.pdf]

Supplementary Material

This PDF file includes Supplementary Figures S1 to S3, Supplementary Table S1 and Captions for Supplementary Movies S1 to S12.

Other supplementary materials for this manuscript include Supplementary Movies S1 to S10.

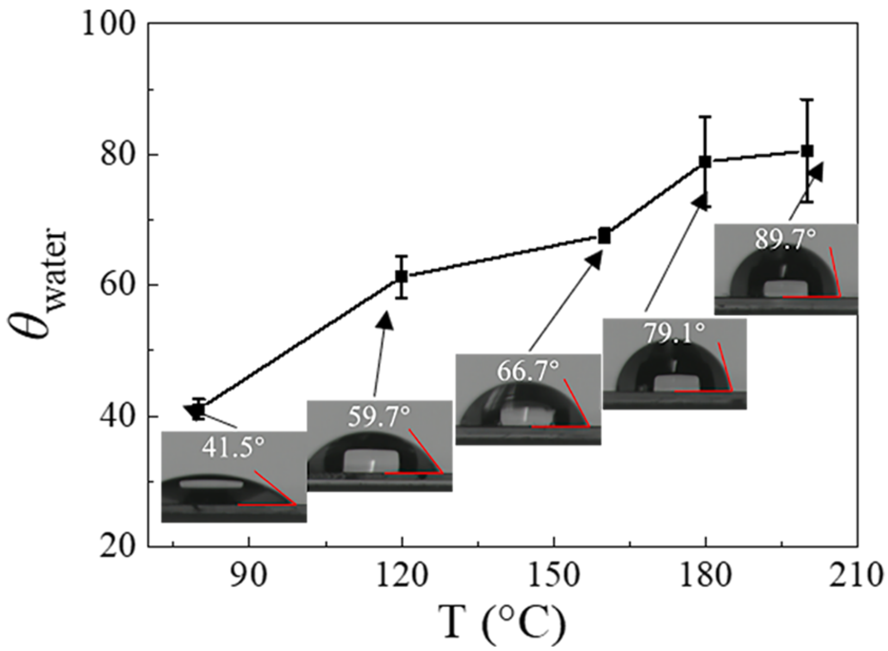

Figure S1. The θ_{water} of PDMS surface treated by plasma and stored in oven for 3h at different temperature (T).

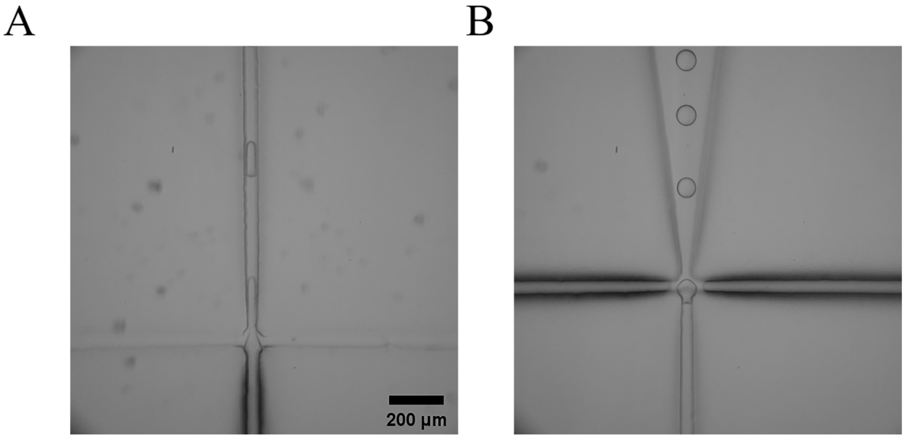

Figure S2. Optical micrograph images showing the unsuccessful formation process of double emulsion droplets at the first junction (A) and second junction (B) with the double emulsion chip without Aquapel treatment. Scale bar, 200 μm.

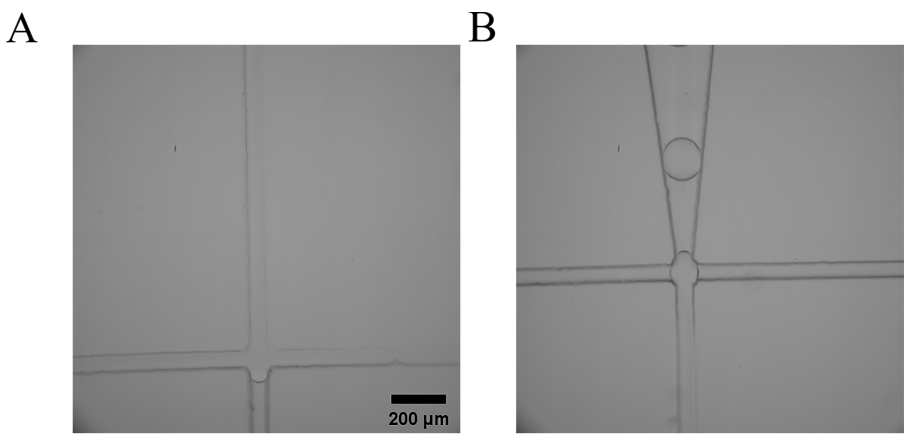

Figure S3. Optical micrograph images showing the unsuccessful formation process of double emulsion droplets at the first junction (A) and second junction (B) with Aquapel treatment. Scale bar, 200 μm.

Table S1. Tested bonding parameters between PDMS replicas and glass slides. Note: ✗, bonding failure; ✓: bonding success. Air flow rate is 60 mL/min.

Treatment\Time	10 s	20 s	30 s	40 s	50 s	60 s	90 s	180 s
30 w	✗	✓	✓	✓	✓	✓	✓	✓
50 w	✓	✓	✓	✓	✓	✓	✓	✗
70 w	✓	✓	✓	✓	✓	✓	✓	✗
90 w	✓	✓	✓	✓	✓	✓	✓	✗
110 w	✓	✓	✓	✓	✓	✓	✓	✗
136 w	✓	✓	✓	✓	✓	✓	✓	✗
150 w	✓	✓	✓	✓	✓	✓	✓	✗
200 w	✓	✓	✓	✓	✓	✓	✓	✗

Table S2. The θ_{water} and θ_{oil} of PDMS surface after Aquapel and PFDTES treatments stored for different time.

Treatment\Time	0 d	3 d	7 d	14 d	21 d	28 d	35 d
Aquapel treatment	102.0±4.7°<br>74.1±3.9°	93.5±3.2°<br>64.2±8.5°	101.8±5.0°<br>61.8±4.6°	93.2±4.2°<br>58.8±3.3°	97.3±2.4°<br>60.0±9.5°	94.2±3.4°<br>64.9±1.2°	93.6±0.5°<br>57.2±3.8°
PFDTES treatment	102.3±5.6°<br>78.4±1.7°	97.6±1.4°<br>75.4±7.5°	96.4±2.7°<br>78.9±1.6°	97.0±0.9°<br>79.2±1.1°	101.8±3.9°<br>77.9±2.5°	94.3±1.6°<br>69.2±6.7°	96.8±4.5°<br>76.6±3.9°

Supplementary Movie Captions:

Supplementary Movie S1. Time-lapse imaging showing the w/PDMS/w double emulsion droplets generation process at the first flow-focusing junction using Aquapel-treated microfluidic device. The movie was taken at a frame interval of 5 ms for 200 ms and was played back at 5 frames per second (fps).

Supplementary Movie S2. Time-lapse imaging showing the w/PDMS/w double emulsion droplets generation process at the second flow-focusing junction using Aquapel-treated microfluidic device. This movie was taken at a frame interval of 5 ms for 200 ms and was played back at 5 fps.

Supplementary Movie S3. Time-lapse imaging showing the generated w/PDMS/w double emulsion droplets in the outlet channel using Aquapel-treated microfluidic device. This movie was taken at a frame interval of 5 ms for 200 ms and was played back at 5 fps.

Supplementary Movie S4. Time-lapse imaging showing the droplets generation process in microfluidic device without Aquapel treatment. This movie was taken at a frame interval of 100 ms for 9 s and was played back at 10 fps.

Supplementary Movie S5. Time-lapse imaging showing the droplets generation process in microfluidic device treated completely by Aquapel. This movie was taken at a frame interval of 100 ms for 9 s and was played back at 10 fps.,

Supplementary Movie S6. Time-lapse imaging showing the w/PDMS/w droplets generation process at the first flow-focusing junction with the Aquapel-treated microfluidic device stored in air for 10 days. This movie was taken at a frame interval of 1 ms for 100 ms and was played back at 10 fps.

Supplementary Movie S7. Time-lapse imaging showing the w/PDMS/w droplets generation process at the second flow-focusing junction with the Aquapel-treated microfluidic device stored in air for 10 days. This movie was taken at a frame interval of 1 ms for 100 ms and was played back at 10 fps.

Supplementary Movie S8. Time-lapse imaging showing the o/w/o double emulsion droplets generation process at the first flow-focusing junction using Aquapel-treated microfluidic device. This movie was taken at a frame interval of 1 ms for 200 ms and was played back at 20 fps.

Supplementary Movie S9. Time-lapse imaging showing the o/w/o double emulsion droplets generation process at the second flow-focusing junction using Aquapel-treated microfluidic device. This movie was taken at a frame interval of 1 ms for 200 ms and was played back at 20 fps.

Supplementary Movie S10. Time-lapse imaging showing the o/w/o double emulsion droplets generation process in the outlet channel using Aquapel-treated microfluidic device. This movie was taken at a frame interval of 1 ms for 200 ms and was played back at 20 fps.

Supplementary Movie S11. Time-lapse imaging showing the w/PDMS/w double emulsion droplets generation process at the first flow-focusing junction using PFDTES-treated microfluidic device. This movie was taken at a frame interval of 1 ms for 100 ms and was played back at 10 fps.

Supplementary Movie S12. Time-lapse imaging showing the w/PDMS/w double emulsion droplets generation process at the second flow-focusing junction using PFDTES-treated microfluidic device. This movie was taken at a frame interval of 1 ms for 100 ms and was played back at 10 fps.
